# Retraction: Genome-wide identification of RETINOBLASTOMA RELATED 1 binding sites in *Arabidopsis* reveals novel DNA damage regulators

**DOI:** 10.1371/journal.pgen.1012022

**Published:** 2026-01-20

**Authors:** The PLOS Genetics Editors

After this article [1] was published, the corresponding author contacted the journal to raise concerns about the integrity and reliability of the results in [1], specifically regarding image overlap in panels describing different experimental conditions in Fig 9C of [1] and Fig EV1B of [2] (withdrawn in [3]), and to request that the journal conduct a further assessment of the images in the article. PLOS noted that in Figs 7 and 9, the following panels appear to partially or fully overlap:

- In Fig 7A:
  - The WT/MS and the +CiPt/*at2g45460-1* panels.
  - The MS/*flip-3* and MS/*ubp21-1* panels.
  - The MS/*kno1-1* and +CiPt/WT panels.
- In Fig 9C:
  - The WT/Mock and *ubp21-1*/Mock - α-γH2AX panels, when levels are adjusted to visualize the background.
  - The WT/Mock and *ubp21-1*/Mock - α-RAD51 panels, when levels are adjusted to visualize the background.
  - The WT/Mock panels and the WT/Mock panels in Fig EV1B of [2] (withdrawn in [3]).
  - The *kno1-1*/Mock panels and the *kno1*/Mock panels in Fig EV1B of [2] (withdrawn in [3]).
  - The *kno1-1*/+CiPt - DAPI panel and the *kno1*/CiPt - DAPI panel in Fig EV1B of [2] (withdrawn in [3]).
  - The *ubp21-1*/+BLM panels and the WT/MMC panels in Fig EV1B of [2] (withdrawn in [3]).

The corresponding author agreed with the above image concerns and noted that the data underlying Figs 7, 8, 9C, and S10-S12 are no longer available. They also stated that they consider that the ChIP data and the article’s main conclusion remains valid, including the claim that these data could be used to identify new DNA damage regulators, and that original data are available to support the article’s other results (Figs 1-6, 9A-B, Figs S1-S9 and S13, Tables S1-S5, Appendices S1 and S2).

In light of the above unresolved concerns about Figs 7 and 9, the *PLOS Genetics* Editors retract this article.

DB, MH, HH, FR, CG, and AS agreed with the retraction. PC either did not respond directly or could not be reached.
